# Trust and mindreading in adolescents: the moderating role of social value orientation

**DOI:** 10.3389/fpsyg.2015.00965

**Published:** 2015-07-21

**Authors:** Jeffrey Derks, Manon A. Van Scheppingen, Nikki C. Lee, Lydia Krabbendam

**Affiliations:** ^1^Department of Educational Neuroscience and Research Institute LEARN!, Faculty of Psychology and Education, VU University AmsterdamAmsterdam, Netherlands; ^2^Department of Developmental Psychology, Tilburg School of Social and Behavioural Sciences, Tilburg UniversityTilburg, Netherlands

**Keywords:** adolescence, trust, mindreading, social value orientation, social development

## Abstract

In adolescence, aspects of cognition that are required to deal with complex cooperation situations, such as mentalising and social value orientation, are still in development. In the Trust Game, cooperation may lead to better outcomes for both players, but can also lead to exploitation by the trustee. In the present study, we explore how mindreading, a crucial aspect of mentalising, and social value orientation (whether someone is prosocial or proself) are related to trust. In a group of 217 students (51% girls, Mage = 15.1) social value orientation, mindreading and trust (using the Trust Game) were measured. The result show that social value orientation moderates the relation between mindreading and trust. In the group of prosocials, we find no correlation between mindreading and trust. In the group of proselfs, mindreading is negatively correlated to trust, indicating that proselfs use their mentalising skills to assess that the trustee is likely to exploit them.

## Introduction

People differ considerably in their motives underlying decision making in situations in which self-interest is at odds with collective interest (i.e., social dilemmas). Some people focus on getting the best out of the situation for themselves, while others try to make sure that all people involved get a fair share (Charness and Rabin, 2002; Fehr and Fischbacher, 2002). These motives reflect proself vs. prosocial value orientations, respectively. These different social value orientations can also be identified during adolescence. However, in adolescence, performance in social dilemmas also depends on ongoing maturation of the social cognitive skills underlying social decision-making (Blakemore and Choudhury, 2006; Van den Bos et al., 2010, 2011a; Fett et al., 2014). The present study investigates how social cognitive skills in adolescence interact with social value orientation (SVO) during decision-making in social dilemmas.

An example of a social dilemma is the Trust Game, originally developed by Berg et al. (1995). In this game, cooperation can lead to an increase in outcome for the self, at the risk of losing the investment. The first player (the trustor) can choose to give a certain amount of money to the second player (the trustee). This amount is tripled, and then the trustee can choose to give a certain amount back to the trustor. Because the amount sent to the trustee is tripled, the trustor can earn more money if the trustee reciprocates, but there is a risk that the trustee will defect. The amount sent by the trustor is used as a measure of trust, and amount returned by the trustee as a measure of trustworthiness. Every trusting interaction involves a risk that the expected reciprocation (trustworthiness) will not take place or will be less than expected. Trust and trustworthiness are basic elements underlying successful cooperative interactions.

In the last decade several studies using the Trust Game have been performed to investigate the development of trust and trustworthiness throughout adolescence. These studies report an increase with age in both trust and trustworthiness from childhood to adolescence (Sutter and Kocher, 2007; Van den Bos et al., 2010, 2011a). The findings on the development of trust and trustworthiness from adolescence to adulthood are less consistent. Most studies find an increase in trust (Sutter and Kocher, 2007; Van den Bos et al., 2011a; Fett et al., 2012), but a decrease has also been reported (Van den Bos et al., 2010). For trustworthiness, most studies report no differences between adolescents and adults (Sutter and Kocher, 2007; Van den Bos et al., 2010, 2011b), but one study finds more trustworthiness in adults (Belli et al., 2012).

In the Trust Game, the trustor's decision on how much money to send to the trustee depends, among other factors, on the trustor's SVO (Kanagaretnam et al., 2009). SVO is often measured using a simple task in which points are distributed between the self and another (Van Lange et al., 1997b). Generally, two types of SVO's are identified: the prosocial and the proself type. Proselfs strive to maximize their own outcome, whereas prosocials strive to maximize joint outcomes and equality of outcomes (Van Lange, 1999). Sometimes the proself type is subdivided in the individualist and competitive type (Van Lange et al., 1997b). Individualists strive to maximize only their own outcome, with no consideration for the outcomes of others. Competitors strive to maximize their own outcome relative to others' outcomes (Bogaert et al., 2008). Since prosocials are inclined to cooperate it can be presumed they are more trusting and trustworthy than proselfs. Some studies have indeed found that prosocials have higher levels of trust and trustworthiness than proselfs (Snijders, 1996; Kanagaretnam et al., 2009).

However, in the Trust Game, as opposed to many other social dilemmas, cooperation may lead to a better pay-off for the trustor than defection. If the trustor presumes that the trustee will return a fair amount of money, the trustor is better off trusting than not trusting. So even proself players, aiming to maximize their own outcome, could decide to cooperate, dependent on their evaluation of the trustee's decision. One way to predict the trustees' decision is by imagining what decision you would make in their place. However, in order to do so, the trustor needs advanced mentalising skills to understand the other's mental processes. Recent studies have shown that more advanced applications of mentalising are still developing until late adolescence (Moriguchi et al., 2007; Dumontheil et al., 2010). The current study focused on the ability to read the mental states of others in their eyes, often referred to as mindreading (Baron-Cohen et al., 1997), as a measure of mentalising or theory of mind. The neural mechanisms of mindreading are still developing throughout adolescence (Gunther Moor et al., 2012; Overgaauw et al., 2014).

The effect of trying to understand the trustee's intentions may be different for prosocial and proselfs. Mindreading may help prosocials to realize that the Trust Game offers a possibility to increase outcomes for both players. Prosocials with good mindreading skills may therefore be more inclined to trust. For proselfs on the other hand, reading the mind of the trustee may reveal that the trustee has the chance to exploit the trust of the trustor. Therefore, proselfs with good mindreading skills may be less inclined to trust. Thus, it can be hypothesized that the relation between mindreading skills and trust is positive in prosocials and negative in proselfs. The aim of the present study was to test this hypothesis. A group of adolescents performed a Trust Game, an SVO task and a mindreading task. The hypothesis was that the relation between mindreading and trust is moderated by SVO.

## Methods

### Participants

In this study, 217 secondary students (*M* age = 15.11 years, *SD* = 0.44, age range = 14.1–16.4) participated of which 111 (51%) were females. All subjects were in the third year of the Dutch secondary education, which can be compared with the 10th grade in the United States. Participants were from five different schools, located in various towns and cities in the Netherlands. Five participants (2%) were excluded from the original sample, because they did not follow the instructions of the test administers. Fifteen participants (7%) were excluded because they failed to complete all measures. The Dutch educational system is subdivided in several levels: secondary vocational education (VMBO), general secondary education (HAVO), and pre-university secondary education (VWO). In the final sample of 197 participants, 92 were enrolled in the VMBO level (47%), 67 in the HAVO level (34%), and 38 in the VWO level (19%). The Trust Game results of the same sample of students are also reported in another paper focussing on the interaction between gender and SVO (Derks et al., 2014).

### Materials

#### Social value orientation

To measure SVO, the Triple-Dominance Measure was used (Van Lange et al., 1997b). This is a paper-and-pencil task that has been tested for real-life validity in several experiments relating a prosocial value orientation to real-life prosocial outcomes (Van Lange et al., 1997a, 2007; De Cremer and Van Lange, 2001; Nauta et al., 2002). The task consists of nine choices that the participants have to make and which influence the outcomes of both the participant and an unknown, hypothetical other. The participant can always choose between three different distributions of points: a cooperative outcome (in which the participant and the other get the same amount of points), an individualistic outcome (in which the participant gets more points than the other) and a competitive outcome (in which the participant gets less points than in the individualistic, but gets more points relative to the other).

When participants made six or more congruent choices they were classified as cooperators, individualists or competitors. As is often done (e.g., De Cremer and Van Lange, 2001; Joireman et al., 2001; Stouten et al., 2005), the individualists and competitors were classified into one group called proselfs (61 participants, 39%), whereas the cooperators are known as prosocials (94 participants, 61%). Forty-two participants (21%) did not make consistent choices and could not be classified. Their scores were not used in the analyses.

#### Mindreading

To measure mindreading skills, the child's version of the Reading the Mind in the Eyes Task (RME) was used (Baron-Cohen et al., 1997, 2001a,b). In this task, the participants are shown a series of pairs of eyes. For each pair of eyes, the participants have to choose the correction option from four listed states of mind. Participants get one point for each correct choice. The task was shortened down from 28 to 15 pairs of eyes due to time restrictions. The response options were translated from English to Dutch. Although different version of the RME have been tested for validity and psychometric properties (Hallerbäck et al., 2009; Fernández-Abascal et al., 2013; Vellante et al., 2013), our Dutch version has not yet been validated.

The RME was originally designed as a measure of Theory of Mind (Baron-Cohen et al., 1997). Several different authors have given different names to the outcomes of the measure such as emotion recognition (Guastella et al., 2010) and social sensitivity (Woolley et al., 2010). In line with a large number of studies (Roeyers et al., 2001; Domes et al., 2007; Hysek et al., 2012; Schilling et al., 2012), we consider the task a measure of mindreading.

#### Trust game

The computerized version of the Trust Game that was used in the present study was based on the original task developed by Berg et al. (1995). In the game, participants were told that they were connected to a peer from another school. However, in reality the responses of the second player were based on a computer algorithm. Each participant played a total of 10 rounds, five times in the role of trustor and five times in the role of trustee. The players were told that they were connected to a different player for each round so that the games were one-shot rounds. This is important because one-shot games tend to invoke different strategies than games with repeated rounds with the same player (King-Casas et al., 2005). The participants were instructed that they (and the other players) could not keep the money made in the game but that they should play as if they would. The decision to use non-real players was made because the testing conditions at the schools made the use of real-time interactions with students from other schools virtually impossible. It was made clear to the participants that the game would be played completely open, thus that all decisions made by the players would be visible to the other player.

Before each round as a trustor, the participants first saw a screen saying that the computer was connecting through the internet with a computer at another school. The subjects also saw the first letter of the name of the other player to make sure they understood each round was played with another player. Then, the participants saw on the screen that they were given €6 and had the choice to send €0, €2, €4, or €6 euro to the other player. If they chose not to give any money the round was finished and the final scores were presented (participant €6, other €0). If they chose to give an amount, this amount was tripled and given to the trustee. The participants were then told that the other player was making a decision about returning money. After a few seconds, the decision of the second player and the outcome of the game were revealed (e.g., participant €6, other €12). Hereafter, the next round as a trustor started. The return of the trustee was pre-programmed as a percentage of the received amount and different for each round, but the same for all participants (round 1 = 50%, round 2 = 33 ⅓%, round 3 = 0%, round 4 = 50%, round 5 = 33 ⅓%).

After the five trustor rounds, the five trustee rounds started. Each round started with the message that the trustors were making a decision. After a number of seconds, the amount sent by the trustor was revealed. The amount given by the trustor was pre-programmed and different per round but the same for each participant (round 1 = €4, round 2 = €6, round 3 = €2, round 4 = €6, round 5 = €4; as is custom in the Trust Game the participant received these amounts in threefold). The participant could send an amount back to the trustor by typing in an integer between €0 and the amount received. The final pay-offs were then revealed on screen (e.g., other player €9, participant €9) and the next round started.

In the statistical analysis, the percentage of the total amount sent by the participant when playing as a trustor was used as a measure for trust. The measure for trustworthiness was the percentage of the amount received by the trustor that was returned when the participant played the role of the trustee. In the instructions and the experiment, words like “trust,” “trustworthiness,” “trustor,” and “trustee” were not used.

### Procedure

The schools that were selected for participation sent a letter to all the parents of eligible participants. In this letter, the study was briefly described and the parents were told that they could object to their child participating in the study. The adolescents themselves also had the opportunity to refuse participation. None of the parents or adolescents objected. In line with our standard procedure for testing at schools, the subjects were not paid for participation and could not earn real money in any of the tasks. The ethical committee of the Faculty of Psychology and Education approved the study.

Testing took place in a quiet room in the schools. Participants were tested in groups of six at a time. First the procedure of the Trust Game was explained to the participants verbally by the study administers. Then, two of the participants were asked to play out a round of the game in front of the other subjects under guidance of the test supervisors to further clarify the rules of the game. Thereafter, some questions regarding the set-up of the Trust Game were asked individually to the participants on a laptop. If the subjects made any mistakes, the test administers gave them extra instructions verbally. Then, the participants performed the Trust Game individually on the laptops. Thereafter, the participants completed the mindreading task, the SVO task and some further questions about personal details (sex, age, etc.) on paper.

### Statistical analysis

First, the relations between trust, trustworthiness, mindreading, SVO, sex, and age were established using Pearson's correlations. In addition, *t*-tests were performed to test differences in levels of trust and trustworthiness between prosocials and proselfs and between boys and girls.

To test if SVO is a valid moderator of the relation between mindreading and trust a regression analysis was performed. In the analysis, SVO (0 = prosocial, 1 = proself), mindreading (centered) and their interaction term (SVO x mindreading) were the independent variables and trust was the dependent variable. Age (centered) and sex (0 = boy, 1 = girl) were used as control variables. Simple slope analyses were used to further explore the interaction effect.

## Results

### Descriptive statistics

The descriptive statistics for trust, trustworthiness, mindreading, SVO, sex, and age and their (cor)relations are depicted in Table 1. Trust was positively correlated with trustworthiness [*r*_(153)_ = 0.33, *p* < 0.001] and this correlation remained significant when controlling for age and sex [*r*_(153)_ = 0.32, *p* < 0.001]. Mindreading was not significantly correlated with trust [*r*_(153)_ = −0.09, *p* = 0.29] or trustworthiness [*r*_(153)_ = 0.12, *p* = 0.14] and these correlations remained non-significant when controlling for age and sex [trust: *r*_(153)_ = −0.07, *p* = 39, trustworthiness: *r*_(153)_ = 0.11, *p* = 0.20]. Prosocials were significantly more trusting [*t*_(153)_ = 3.47, *p* = 0.001] and more trustworthy [*t*_(153)_ = 4.47, *p* < 0.001] than proselfs. Furthermore, boys were more trusting than girls [*t*_(153)_ = 2.43, *p* = 0.02], but there were no significant sex differences in trustworthiness [*t*_(153)_ = −0.67, *p* = 0.51]. Moreover, age was a negatively correlated to trust [*r*_(153)_ = −0.19, *p* = 0.02] and trustworthiness [*r*_(153)_ = −0.21, *p* = 0.008]. Please note that these results have already been published and discussed in Derks et al. (2014).

**Table 1:** **Descriptive statistics and Pearson correlations**.

	***M***	***SD***	**1**	**2**	**3**	**4**	**5**
Trust	48.4%	17.9%	–				
Trustworthiness	31.1%	13.5%	0.330^**^	–			
Mindreading	9.10	2.05	−0.086	0.119	–		
SVO score (0 = prosocial, 1 = proself)	0.39	0.49	−0.270^**^	−0.340^**^	−0.125	–	
Sex (0 = male, 1 = female)	0.52	0.50	−0.139^*^	0.054	0.150	−0.155	–
Age	15.12	0.45	−0.185^*^	−0.214^**^	−0.061	0.131	−0.102

* *p < 0.05*;

** *p < 0.01*.

### Moderator analyses

**Figure 1:**
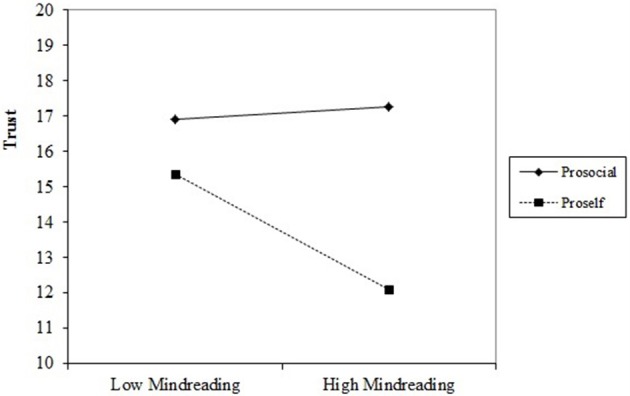
**Plots of slopes for the interaction between mindreading and social value orientation (prosocial or proself) on trust, using sex and age as control variables**. In the Plot, low mindreading is set at −1 standard deviation from the mean and high mindreading at +1 standard deviation.

Regression analyses were performed to test the moderating effect of SVO on the relation between mindreading and trust. In the first step, SVO, mindreading and the two control variables, age and sex, were added as predictors. In the second step, the interaction between SVO and (centered) mindreading was added as a dummy variable. The results of the regression analyses are depicted in Table 2. The R^2^-change between model 1 and model 2 was significant (${\text{R}}_{\text{change}}^{2}=0.026$, *p* = 0.029). The interaction term in model 2 was significant [β = −0.88, *t*_(149)_ = −2.21, *p* = 0.029] indicating that SVO was a significant moderator of the relation between mindreading and trust. To further investigate the nature of this moderation, simple slope analyses were performed separately for the prosocials and proselfs. These analyses revealed that in the group of prosocials there was no significant relation between mindreading and trust [B_mindreading_ = 0.09, *t*_(93)_ = 0.36, *p* = 0.72]. However, for the proselfs the effect of mindreading on trust was significant [B_mindreading_ = −0.79, *t*_(60)_ = −2.55, *p* = 0.01], indicating better mindreading skills were associated with lower trust. The slopes of this interaction are plotted in Figure 1.

**Table 2:** **Summary of regression analysis for variables predicting trust without interaction term (Model 1) and with interaction term (Model 2)**.

**Variable**	**Model 1**			**Model 2**		
	**B**	**SE B**	**β**	**B**	**SE B**	**β**
Social value orientation	−3.251	0.836	−0.297^***^	−3.363	0.827	−0.307^***^
Mindreading	−0.257	0.199	−0.098	−0.089	0.251	0.034
Age	−2.124	0.906	−0.177^*^	−1.992	0.896	−0.166^*^
Sex	−2.593	0.817	−0.242^**^	−2.596	0.807	−0.242^**^
SVO × Mindreading				−0.883	0.399	−0.210^*^
R^2^		0.168			0.194	

* *p < 0.05*;

** *p < 0.01*;

*** *p < 0.001*.

## Discussion

In the present study a group of adolescents performed a Trust Game, a mindreading task, and an SVO measure. Adolescents with prosocial preferences were more likely to trust than adolescents with proself preferences. The results further reveal that SVO moderates the relation between mindreading and trust in adolescence. Within the group of proselfs, mindreading skills were negatively related to trust indicating that those with good mindreading skills were less likely to trust than those with worse mindreading skills. It seems that having an understanding of the mind of the others makes proselfs less likely to risk their money in the Trust Game. Within the group of prosocials, a positive correlation was expected. However, for these adolescents mindreading was unrelated to trust.

A possible explanation for the finding that the moderation is driven by the negative association between mindreading and trust within the proselfs may be that prosocials and proselfs frame social dilemma decisions differently. Prosocials tend to frame decisions in terms of morality (moral or immoral decisions), whereas proselfs frame them in terms of power (weak or strong decisions) (Liebrand et al., 1986; Bogaert et al., 2008; Balliet et al., 2009). For prosocials, reading the mind of the other may not influence the decisions since they view cooperation as a moral obligation, regardless of the decision of the other player. For proselfs, the trust decision is influenced by their their ability to mentalise because they may view the decision to trust as weak when they realize that they can easily be exploited. In addition, prosocials and proselfs may differ in their assumptions of others' preferences. The *triangle hypothesis* (Kelley and Stahelski, 1970; Van Lange, 1992; Iedema and Poppe, 1995; Weingart et al., 2007) states that prosocials hold a heterogeneous view of the preferences of others, assuming that others are either prosocial or proself. On the other hand, proselfs are thought to hold a more homogeneous view of other's preferences, assuming that most others are proself like themselves. In the Trust Game, proselfs with good mindreading skills may decide not to trust, because they understand that the other can exploit them by not trusting. On the other hand, prosocials with good mindreading skills may realize that the trustee can either repay or exploit the trust decision. Since they have no presumption of the SVO of the trustee, their trust decision may not be affected by their mindreading skills.

Adolescent cooperative behavior is complex and adolescents have to learn to deal with others who may or may not be trustworthy or reliable. In order to predict the cooperation decisions of others, adolescents need to be able to mentalise, a skill that is still developing throughout adolescence (Dumontheil et al., 2010). Recent studies have found that mentalising skills are important in understanding adolescent social decision making (Güroğlu et al., 2009; Van den Bos et al., 2011b; Fett et al., 2014). The present study extends these findings by showing that the effect of mentalising skills is moderated by the prosocial orientation of the adolescent.

A number of limitations of this study have to be mentioned. First, earnings in the Trust Game were not actually paid out to the participants. Payments being hypothetical influences the outcomes of social dilemmas according to some (Hertwig and Ortmann, 2001; Vlaev, 2012), but not all studies (Madden et al., 2003; Locey et al., 2011). In a meta-analysis, Balliet et al. (2009) find that hypothetical payments may influence the strength but not the direction of the relation between social value orientation and cooperation in social dilemmas. Second, in this study mindreading was used to measure mentalising skills. It could be argued that mindreading does not capture all the aspects needed to be able to mentalise. However in this study, we have used mindreading as a proxy for mentalising or theory of mind, based on the original intentions of the authors (Baron-Cohen et al., 1997).

In summary, the results of the present study show that the effect of mindreading skills on trust in a Trust Game is moderated by SVO in adolescence. In prosocials there is no relation between mindreading and trust, whereas in proselfs, higher mindreading was correlated with lower trust. It thus seems that proselfs use their mindreading skills to infer that the trustee might not repay their trust. More research is needed to see if the same effects are found in adult populations, which in general will have better developed mentalising skills. In addition, the role of SVO and mentalising can be studied in cooperation situations other than the Trust Game.

### Conflict of interest statement

The authors declare that the research was conducted in the absence of any commercial or financial relationships that could be construed as a potential conflict of interest.
